# What do university students say about online learning and the COVID-19 pandemic in central Fiji? A qualitative study

**DOI:** 10.1371/journal.pone.0273187

**Published:** 2022-08-23

**Authors:** Eunice Okyere, Mosese Salusalu, Ramneek Goundar, Kissinger Marfoh

**Affiliations:** 1 Department of Public Health, College of Medicine, Nursing and Health Sciences, Fiji National University, Suva, Fiji Island; 2 Department of Public Health, Korle-bu Teaching Hospital, Accra, Ghana; Central China Normal University, CHINA

## Abstract

Globally, the spread of COVID-19 has led to the closure of schools, thereby accelerating the expansion of the online learning environment. Though, Fiji National University students’ (FNU), had no option than to quickly adopt to this mode of learning, within limited period, their learning experiences are yet to be examined and documented. We used phenomenological study design to explore students’ online learning challenges, coping strategies and their perceptions on the causes of COVID-19. A total of 120 in-depth interviews were conducted with FNU students, at different levels and colleges, and analysed thematically, using inductive approach. The three themes emerged included COVID-19 misconception beliefs among students, online learning challenges during the COVID-19 pandemic and online learning coping strategies during the COVID-19 pandemic. The misconception beliefs identified were natural occurrence, manmade for depopulation, unreal/fake and as a means of soliciting for funds. The challenges included ineffective tutorial sessions, lack of learning devices, unstable internet service, inadequate learning environment, socio-cultural practices, feeling of loneliness, anxiety and stress, and difficulties accessing online platforms and acquiring practical skills. The coping strategies used by students ranged from support from family and counsellors, help-seeking, frequent communication, time management, learning flexibility to control over learning environment. The findings highlight the need for policy makers, school managers, lecturers and other key stakeholders to address online learning challenges to improve online learning among FNU students. Relevant information should be provided on the COVID-19 pandemic to clear misconceptions.

## Introduction

The COVID-19 pandemic has affected all levels of the educational systems, ranging from pre-school to tertiary education, subsequently affecting over 1.5 billion students [1]. This has led to the temporal closure of schools, cancellation of face-to-face classes and examinations as well as strictly observing physical distancing. These measures have ignited the digital transformation related to higher education systems, challenging its capability to respond in a prompt and efficient manner. Schools worldwide embraced various forms of technologies, modified resources for learning, developed new protocols for teaching, established systems and infrastructures as well as adjusting their curricula. Whilst this transition was carried out efficiently for some schools, it was not so for others, especially those in low-income countries with inadequate infrastructures [1, 2].

Given the current uncertainties, the world is experiencing significant changes in terms of technology in the education environment, including the expansion and implementation of online learning across various learning settings, either formal or informal. Recently, schools and students worldwide are steadily embracing online learning technologies that enable lecturers to offer instruction in an interactive manner, distribute learning resources as well as facilitating interactions among students [3–5]. Online learning involves the utilization of the internet and other technical devices and tools for synchronous and asynchronous delivery of instructions and managing academic programs [6, 7]. While synchronous online learning comprises, real-time interactions occurring between lecturers and students, asynchronous online learning takes place in the absence of a strict schedule for various students. Thus, asynchronous method of online learning, does not need real-time interactions [8, 9]. Studies have established the effectiveness of online learning, but the challenges associated with its implementation is increasing [10].

Other scholars have highlighted the challenges students face regarding online learning to those related to self-regulation, competency in the use of technology, student isolation, technology adequacy and complexity [10–12]. The self-regulation aspect relates to the ability of students to control their emotional state, actions and opinions to attain their learning objectives. The competency related to technology, has got to do with the capability of students to use technology effectively for the purpose of learning. Students’ isolation refers to the emotional discomfort experienced by students due to loneliness and been separated from their peers or classmates. While technological adequacy relates to various challenges students face in accessing existing online technologies for learning, technological complexity includes challenges faced by students, resulting from their exposure to technologies which are complex and overly sufficient for online learning.

Additional challenges associated with online classes have been identified by researchers in relation to resources [13, 14]. These underpin the possibility of students experiencing challenges related to the utilisation of library and instructional resources as well as the challenges that students experience based on their learning experiences and approaches. The difficulty students’ face in understanding the subject content, during online classes and internet connectivity challenges have been established [15, 16]. Together, these have significant impact on students’ attainment of learning outcomes [17].

Currently, the COVID-19 pandemic has posed a great challenge to the education system in Fiji, which has necessitated governments and policy makers to put in place interventions to minimize the impact of the pandemic on education, such as providing technological learning resources and the modification of curriculum, assessment and methods of instructional delivery. These changes forced schools in Fiji to move to full online learning, of which FNU, is without an exception. Due to the uniqueness of this approach, many students had no option than to quickly adopt to this mode of learning, within limited period. Online teaching and learning have become the order of the day for both lecturers and students worldwide, hindering both parties from enjoying the benefits derived from social interactions or typical learning school settings [18].

The recent situation could increase the challenges students face during online learning, resulting from mobility restrictions and healthcare protocols [19, 20]. As such, it is necessary to explore and understand the experiences of students regarding online learning during the COVID-19 pandemic. Almost all the previous studies examining the general experiences of students, highlighted above, used quantitative methodologies. This study adds to literature by using qualitative approach to specifically, explore the challenges students face during online learning, and their coping strategies in Fiji context. It also explores the perceptions of students regarding the causes of COVID-19, for appropriate interventions. Fiji Island is already battling with challenges related to climate change and natural disasters including cyclones. The COVID-19 has compounded the issues, by revealing the vulnerability of the limited capacity of the healthcare system. These could have adverse effects on students learning ability, hence the relevance of the study. We therefore ask the questions below in that regard:

1. What are the perceived causes of COVID-19 pandemic among students?
2. What are the online learning challenges faced by students during COVID-19 pandemic?
3. What are the strategies used to overcome online learning challenges during the COVID-19 pandemic?

As the first study to be conducted among students in the Fiji Island, it is vital to provide relevant information in this area, specifically, within the context of the COVID-19 pandemic. Thus, revealing relevant information on students’ online learning experiences could enable policy makers, school managers, lecturers and other key stakeholders to provide adequate support to the online learning requirements of students.

## Methods

### Ethical consideration

Ethical clearance for this study was sought from the College Human Health Research Ethics Committee (CHHREC), Fiji Institute of Pacific Health Research (FIPHR), with approval number 298.20. All the protocols used in this study, followed the ethical principles of the university. Relevant and detailed information on the study (information sheet), was given to the study participants with whose concerns and questions were thoroughly addressed. Participants were made to sign informed consent forms prior to all in-depth interviews. For the few interviews conducted on phone or zoom, detailed information regarding the study, including participants rights were read to them, and consent forms emailed to participants for signing. Participants were informed about the voluntarily nature of the study and their right not to respond to a particular question. Participants’ real names were replaced with codes in the audio recording and all identifiers separated from the data to prevent the possibility of being identified.

### Study setting

The study was conducted among FNU students. The university, which is in Suva (the capital), has different colleges with a population of approximately 20,000 students [21]. Participants were selected purposively to reflect diversity across the different colleges, including, College of Agriculture, Fisheries & Forestry (CAFF), College of Business, Hospitality & Tourism Studies (CBHTS), College of Engineering, Science & Technology (CEST), College of Humanities & Education (CHE) and College of Medicine, Nursing & Health Sciences (CMNHS).

### Study design

A qualitative study with phenomenological research design was used to explore students’ online learning experiences during COVID-19 pandemic. Specifically, they were asked to share their views on the causes of COVID-19, their online learning challenges and coping strategies. Thus, the researcher used the experiences of the study participants as the authentic source of knowledge in this study [22].

Qualitative method offers a deeper analysis and provides an in-depth understanding of how respondents explain their situations, and interpret their occurrences [23], thereby allowing individuals to contribute to knowledge. The phenomenological approach has been established to facilitate the interpretation of meanings of phenomena experienced by study participants. This further enhances the understanding of the perceptions of individuals who live an entire phenomenon and make sense of it [24].

The flexibility nature of the method used in this study enabled the researchers to explore and understand the subjective experiences and meanings of the students [24]. More importantly, it enabled the study participants to express and understand their feelings and experiences using their own words [25, 26]. Since it is impossible for researchers to clear their memories of their previous knowledge, this study took into consideration the importance of researchers’ reflexivity [27, 28].

Reflexivity has been proven to be important in ensuring rigor and quality in qualitative research, which allowed the researchers to better apprehend their roles in creating knowledge [27, 28]. It further enabled the researchers to realize that meanings are conveyed within a particular occurrence, context and settings. We, therefore, sustained flexibility through reflexive journal and by taking reflexive notes during the in-depth interviews, which provided in-depth information to enrich the meanings of data collected [29].

### Study participants

This study recruited a sample of 120 students between February to October 2021. Data saturation was reached when there was no new information or themes observed in the data [30]. Participants were identified through relevant authorities within various colleges and contacted directly, through email or phones calls to request their participation. The inclusion criteria for selecting participants demanded them to be a student of FNU, 18 years or above, able to speak English and have no disability like mental disorder or ill health. To enhance diversity in the data collected, factors such as age, gender, tribe, religion and marital status were taking into consideration in choosing participants.

Data collection involved conducting in-depth interviews using a semi-structured interview guide (S1 File), which lasted for 30–45 minutes, and tape recorded. The guide was piloted to ensure the appropriateness of the structure and length [23]. Semi-structured interview has been established to be flexible and this could be modified in the process to improve the trustworthiness of the study [31]. The data collection started with face-to-face interviews at a place respondents regarded comfortable. However, this was changed to online interviews (phone and zoom), when the COVID-19 community transmission began in the Fiji Island. The online data collection was initially in cooperated into the study design to take care of emergency situations. These interviews were carried out at times agreed upon by both the interviewer and interviewee. The researchers ensured that, the data collection created a non-threatening and comfortable environment and provided greater ease for participants discussing sensitive issues [32, 33].

### Data handling and analysis

The audio recordings and field notes were transcribed by the team members, who checked the accuracy and quality of the transcriptions with the audio-records. The thematic analysis approach adopted for this study, was that, proposed by Braun and Clarke (2006). This approach identifies themes and sub-themes that may emerge from the data, thereby, providing rich and detailed description of the data set. Nevertheless, thematic analysis usually goes further by interpreting various facets of the study topic [34]. The six-steps used in the analysis included data familiarizing, generating initial codes, searching for themes, reviewing the themes, defining and naming the themes and finally producing the report.

The analysis began with two of the researchers familiarising and emerging themselves with the data through multiple readings and notes taking. This process of immersion involved reading the data multiple times in an active manner to search for meanings and patterns. During this phase, the researchers began to take notes for coding which informed the subsequent phases. This phase of the analysis was also enhanced through the transcription process, which included writing of verbatim all the verbal and non-verbal words. The researchers ensured that the transcripts retained the information needed in its original state and this further led to deeper understanding of the data. The second phase involved identification of initial codes from the data. Through this, interesting segments of the raw data or information regarding the phenomenon under study were identified, making it possible for the data to be organised into meaningful groups. The initial codes identified were matched together with data extracts showing that code, and all actual data extracts were coded and collected within each code.

The third step of data analysis started after the coding was completed and collected. During this stage, the researchers focused on finding themes at the broader level by organizing various codes into potential themes and collected all the coded extracts within the identified themes that were important. In this case, the researchers after examining the codes, identified those that fitted together into themes. For instance, we had some codes that related to online learning challenges and how students were able to cope with these challenges. In the fourth phase, the researchers reviewed the themes produced through thematic mapping of the analysis and proceeded to check how relevant the themes were from the entire data and the coded extracts, to ascertain if they made sense. At this stage, we gathered all the data important to each theme. Thus, relevant data were collated to each possible theme to assess the quality of the coding [35]. We then examined the extracts collected to determine if the identified themes constituted an essential component of the entire dataset. By this, we were able to ascertain whether the themes identified were true reflection of the dataset, the meaning they conveyed, the link between them and their consistency in the data.

The themes were then defined and refined in phase five. The essence of each theme and the aspect of the data each of them captured were identified. The interactions and relationships of the subthemes to the main themes were identified and the collected data extracts for each theme, organised into a coherent and internally consistent account with accompanying narratives. To minimise bias, the coding and the combination of the codes into themes were carried out mainly by two of the researchers, which were further verified by two additional team members. The emerging themes were refined and analysed further by moving back and forth ‘the part and the whole’ transcribed texts to identify structures of the main theme. Finally, the researchers reviewed and analysed all themes in relation to the research question, and these were used in the write-up report. The thematic mapping diagram shows a link between the themes and sub-themes emerging (S2 File).

The triangulation of narratives from students at different levels and colleges, improved the validity of the data. Data analysis involved inductive approach, where categories and themes were identified from the data [36].

### Rigor and trustworthiness

In establishing trustworthiness in qualitative research, four rigorous criteria has been established: credibility, confirmability, dependability and transferability [37–39]. The interview process and techniques used increased the credibility of this study. Thus, the semi-structured interview procedure permitted flexibility and focus during the in-depth interviews. Starting with broad questions and the possibility to include several probes allowed the researchers to request for additional information, where necessary. Also, investigators authority in the area of study was established [40], to improve the credibility of the study, by ensuring that, the researchers were familiar with phenomenon and the context of the research and those conducting the interviews had experience in collecting qualitative data. The credibility was further improved through the audio-tape recordings of the in-depth interviews to ensure capturing the exact words of participants [23]. The researchers’ reflexive notes combined with the in-depth interviews, enhanced the quality of the data collected [41].

The confirmability criterion was measured by establishing an audit trail. This included, taking steps to keep a track record, the processes involved in the data collection by maintaining communication within the research team to make sure those conducting the interviews abided by the protocol in recruiting study participants. The team involved in the data analysis took a systematic approach to review the transcripts against the audio files for consistency, clarifications and accuracy [42]. The steps involved in coding the data as well as the identification of key concepts, categories, sub-themes and themes were approved by the research team.

The monitoring of the data analysis process enhanced consistency and correct interpretation of results throughout the data analysis [43]. The rich description of the processes involved in the study, including, data collection methods, analysis and interpretation were used to assess the dependability criterion. Regarding transferability criterion, as indicated in the methods section, purposive sampling was used to ensure diversity in terms of selecting participants across the study setting, which made it possible to compare analysis across various groups [39, 44]. The researchers, maintained rigor and trustworthiness throughout this study, including ethical issues.

## Results

Out of the 120 students interviews, majority were males (53%), single (82%) and from the I-Taukei ethnic group (52%). Most of the participants were undergraduates (87%) in the CMNHS (42%) and Christians (50%). The age ranged from 19–30 years (Table 1).

**Table 1:** Demographic characteristics of students interviewed.

Students	n = 120
**Median Age in years (range)**	24 (19–30)
**Sex**	
Male	64 (53%)
Female	56 (47%)
**Ethnicity**	
I-Taukei	62 (52%)
Indo-Fijian	51 (43%)
Others	7 (5%)
**Marital status**	
Single	98 (82%)
Married	22 (18%)
**Religion**	
Christian	60 (50%)
Hinduism	53 (44%)
Moslem	7 (6%)
**Programme level**	
Master	10 (8%)
Bachelor	87 (73%)
Diploma	18 (15%)
Certificate	5 (4%)
**College**	
Medicine, Nursing, Health Sciences	50 (42%)
Engineering, Science, Technology	22 (18%)
Agriculture, Fisheries, Forestry	27 (23%)
Business, Hospitality, Tourism	10 (8%)
Humanities, Education	11 (9%)

Inductive analysis of the data resulted three broad themes and subthemes. The themes included COVID-19 misconception beliefs among students, online learning challenges during COVID-19 and online learning strategies during COVID-19 (Table 2).

**Table 2:** Themes and sub-themes.

Themes	Sub-themes
COVID-19 misconception beliefs among students.	Natural occurrence
	Manmade for depopulation
	Unreal/fake
	Means of soliciting for funds
Online learning challenges during the COVID-19 pandemic.	Ineffective tutorial sessions
	lack of learning devices
	Unstable internet access
	Difficult accessing online learning platforms
	Feeling of loneliness
	Anxiety and stress
	Inadequate learning environment
	Difficult acquiring practical skills
	Socio-cultural practices
Online learning coping strategies during the COVID-19 pandemic.	Learning flexibility and comfort
	Easy assessment procedure
	Family support
	Counselling support
	Help-seeking
	Frequent communication
	Time management
	Control over learning environment.

Presented below are the findings under the themes and sub-themes together with explanatory statements from the study participants.

### COVID-19 misconception beliefs among students

#### Natural occurrence

Most of the students viewed COVID-19 as a natural occurrence, influenced by God. As such, it should be accepted as the will of God for mankind, instead of blaming humans for causing the pandemic. A 22-year-old student explained accordingly,

*“I will say that COVID-19 is something that has happened naturally*, *and we cannot put the blame on anybody*. *It’s difficult to explain some of these things so I will say God knows it all*. *As human beings we will talk about virus causing COVID-19*, *which is ok*, *but this is not straight forward*. *Is like every 100 years something like this happens*. *I learnt about 100 years ago*, *there was another pandemic that wiped people from the surface of the earth*. *This is sad but only God knows best”*. (P70)

#### Manmade for depopulation

Some of the participants described COVID-19 as a scam caused by secret societies to reduce human population worldwide and put fear in people. This was what one of the students had to say during the in-depth interviews,

*“In my point of view*, *I believe COVID-19 was the biggest scam of all times*. *It was a manmade virus*, *created by the secret societies for their depopulation agenda and to put fear in humans*. *For some years now*, *I have been researching about the secret society*. *They are known to many as the illuminati or the Free Mason*, *but these are just small societies who dance to the tunes of the elites*. *So*, *from there*, *I learnt of their agenda and one of it is the virus that’s affecting everyone in the world today*. *If you are not awake than you won’t believe what I’m telling you”*. (P7)

#### Unreal/Fake

The existence of COVID-19 was questionable among some of the students since many didn’t believe it was real. Instead, the awareness creation, was considered as fake news, propagated through the media by the elites in the society, to put fear in human beings,

*“If you ask my friends about my view towards this plandemic or scamdemic*, *or for the coronavirus*, *they will tell you that I think that it’s all fake*. *So COVID-19 was planned to happen many years ago and the elites are those spreading the news in the media*. *You should try and watch the opening ceremony of the 2012 Olympics*. *The elites have a lot of things to do*, *and this pandemic is the beginning of it*. *Worse is yet to come”*. (P20)

#### Means of soliciting for funds

It appeared COVID-19 had taken a political dimension in the study area since some of the students, who didn’t believe in its existence, explained that the government was deliberately spreading the news in order to get foreign assistance or aids in monetary form. A student, aged, 25 years explained accordingly,

*“To be honest*, *I just continue with my normal routines*. *I believe that the Fiji government is just manifesting what the people want*. *I believe there is no virus in Fiji right now*. *This is all just a show*, *to get aid from overseas*, *so they can make money*. *There is no Indian variant here*. *Everyone is a joke”*. (P17)

Another respondent in support of what the above respondent said, believed, the government was throwing dust into the eyes of the citizens and condemned the media for spreading false information on COVID-19,

*“I feel sorry that we are being used as a scape goat because I don’t believe there is any virus*. *The government is using it to get money from developed countries*. *The time is coming when people will know the truth*, *and the worst part is the media*, *the biggest liar*, *making us believe in what they tell us*, *which is not true”*. (P34)

While some of the participants considered COVID-19 to be intentionally planned by humans to reduce the world’s population, others didn’t believe it was real. The God factor came up strongly among those who regarded COVID-19 as a naturally occurring phenomenon, with others viewing it as a source of fund raising for the government.

### Online learning challenges during the COVID-19 pandemic

#### Ineffective tutorials session

Students described their online discussions and tutorials as difficult, boring and tiring. This was largely observed among those who had just transited from face-to-face classes to online. Many had not come to terms with the need for the restrictions put in place by the government to control the spread of COVID-19,

*“Yes*, *COVID-19 has affected my usual way of learning*. *I am now having online classes*, *which is difficult for me especially attending discussions and tutorials*. *I feel tired on a day-to-day basis and bored sometimes*. *It is a lot of work learning online but yes*, *these restrictions by the cruel and greedy government are affecting every student in Fiji and there is nothing we can do about it”*. (P3)

Most of the students preferred face-to-face tutorials to online. In support of what P3 said above, another student explained the challenges she encountered in attending tutorial sessions and recommended for lecturers and other key stakeholders to improve the situation.

*“I think our lecturers and the people running the school should do something about online tutorials and make it enjoyable like the face-to-face tutorials*. *I don’t like the online tutorials at all*. *It’s very boring and not interesting*. *I learn a lot from the face-to-face tutorials but now everything is online*, *and things are not working properly*. *Is a big problem but what can we do*. (P56)

#### Lack of learning devices

One of the major frustrations expressed by most of the students was lack of appropriate devices (phones, ipad and computers) for online learning. This was attributed to financial constraint, resulting from jobs lost during the COVID-19 crisis. In attempt to salvage the situation, students borrowed laptops from their friends and relatives, which sometimes affected their online learning pattern and flow,

*“I don’t have a phone at the moment so it’s hard keeping up with zoom lectures*. *I don’t also have a computer or iPad*. *There is no money to buy a computer since my father lost his job because of COVID so tell me how a student will be able to engage in online classes without these things*. *I borrow from my friend or cousin*, *but I can only get it when they don’t have a class*…*”* (P85)

The possibility of students missing their online classes was high among those who depended on others for online learning devices. The challenge usually arises when the two individuals have scheduled online classes at the same time,

*“It is very difficult keeping up to date with discussions and tutorials plus zoom lectures*. *Sometimes*, *my brother’s classes clash with mine*, *so I just sacrifice because it is not my laptop*. *So*, *I can say my challenge is not having a phone and a laptop*. *It is very hard for me*. *I find it easier with face-to-face learning”*. (P73)

#### Unstable internet service

Reliable internet is important for online learning. In the absence of a stable internet, attending online classes and accessing reading materials become nearly impossible. One of the respondents explained the situation,

“*It’s like a first class learning and although I feel for the students who cannot afford data and sometimes too the internet is not good*, *so it goes on and off during online classes… When the internet becomes slow*, *you cannot even download articles and books to read”*. (P15)

#### Difficult accessing online platforms

Participants during the discussions, expressed concerns about access to online learning platforms. They explained the difficulties they sometimes encountered in accessing reading materials from the university’s library, submitting their assignments and assessing their online quizzes,

*“I will say the COVID-19 is causing more harm to students because it has made learning very difficult*. *This is why I like the face-to-face classes but because of COVID-19*, *we are all doing online learning and this is too difficult*. *Sometimes I even find it difficult to access my online quiz and upload assignments on Moodle and also when I am searching for papers and books from the library online…”* (P9).

#### Feeling of loneliness, anxiety and stress

Majority of the students’ expressed the feeling of been alone and emotionally disconnected from their peers as a major challenge related to online classes. Meeting their peers on campus increased their sense of belonging amid other things as receiving learning and emotional support,

*“The truth is that I don’t feel part of my friends during online classes*. *You just feel like you are alone and isolated from everybody*. *That connection is not there so I am not interested in online classes at all*. *As a student you just want to meet with your friends on campus and feel that you are also part of them not online*. *Before the COVID*, *we sometimes shared ideas during group discussions*, *and we were there for each other in many ways*, *but we cannot do that now”*. (P36)

#### Inadequate learning environment

Students didn’t find their homes suitable for online learning due to distractions. Many couldn’t afford to rent their own apartment due to financial constraint, and as such, shared rooms with their relatives. This made it difficult to control distractions such as noise and other interruptions from their relatives. They further explained how this has served as a limitation to the timely completion of their assignments and other course requirements,

*“I know some of my friends are happy learning at home*, *but I am not happy because I am not able to focus very well*. *The best thing is for me to rent my own apartment*, *but I have no money for that so I’m sleeping in the room with my two sisters*. *Sometimes I don’t even get the time to sit and work on my assignments and do my online quizzes on time”*. (P49)

Students who were unable to prioritize their activities and manage their time efficiently usually missed their online classes, as explained below,

*“When you are home*, *you get many distractions such that you don’t even know when is the good time and place to sit and learn*. *Sometimes*, *I work in my farm and after that get tired that I even forget to read and miss my online classes*. *I will say home is not the good place to learn because when I go to campus*, *I can sit in the library and other places to read and if I don’t understand anything*, *I can talk to some of my lecturers and classmates”*. (P83)

#### Difficult acquiring practical skills

Many students, particularly those undertaking practical oriented courses expressed their frustrations related to their inability to engage in their usual learning practicals such as fieldwork, laboratory test and internships. They acknowledged the positive effects of these activities, which online learning cannot adequately provide,

*“I will say the online learning has many challenges*, *affecting students*. *Just consider somebody like me who is taking courses that need fieldwork*. *How can I complete my studies in agriculture without going to the farms to get practical experience*? *So*, *you will agree with me that the online learning is not helping us at all but what can we do now that COVID-19 has taken over the system”*. (P97)

*“The main challenge we are facing as students doing health courses is that we are not able to do practicals like lab tests and go for internships*. *These things are not possible because COVID-19 has restricted our movements throughout the country*, *but I learn a lot during practical sessions*. *Online learning is good*, *but it can only provide theories not practicals…”* (P55).

#### Socio-cultural practices

The Fiji community largely practices the extended family system where both the nuclear and extended families usually come together to celebrate their festivals and engage in some religious activities. Though the COVID-19 restrictions imposed by the government, minimised the frequency of these gathering, some families still met to observe their cultural practices and religious activities, which affected students in their studies.

*“I will say that the religious activities going on in my house is affecting my studies and I cannot do anything about it because my father leads people to pray and perform other rituals at home for protections and other things*. *Few weeks ago*, *we came together to celebrate our festival*. *Now because of the COVID restrictions many people don’t come to the house to pray but the extended family members and close friends come over and they make a lot of noise*, *which makes it difficult for me to attend online classes*. *Apart from the noise*, *I have to also attend these events*, *so I sometimes miss my classes*. (P18)

The online learning challenges experienced by students were difficulties in accessing online platforms, attending discussions and tutorials and acquiring practical skillset. The absence of online learning devices, such as computers and internet services and the distractions at home were additional challenges. Mental health related issues such as anxiety, loneliness and stress were identified among students as well as socio-cultural practices causing distractions.

### Online learning coping strategies during the COVID-19 pandemic

#### Learning flexibility and comfort

Most of the students regarded online learning as flexible and comfortable mainly because they were able to engage in other activities or relax during lectures, by switching off the cameras on their devices,

*“…I think it is a good strategy*, *getting students to engage in online lectures*, *tutorials and more*. *Students are enjoying it because some just turn off their cameras from home and just listen to the lecture while having breakfast or lying down*. *I sometimes do other things in the house like cooking and at the same time listening to my lecture”*. (P22)

#### Easy assessment procedures

Most of the students preferred the online exams conducted compared to the traditional way of conducting examination, due to the absence of invigilators to monitor the examination process. With such flexibility, students sometimes searched online sources for answers,

*“One good thing about COVID-19 is that*, *now we don’t write normal exams like sitting in a big hall with somebody watching over us so that we don’t copy from each other or make noise*, *which is difficult*. *Now it’s easy because everything is online*. *You can easily copy the answers of your test questions from google*, *which makes things easy for students”*. (P23)

Although students preferred to engage simultaneously in other tasks during online classes as a means of minimizing loneliness and depression, they also admitted to this practice causing a distraction, thereby minimizing their focus and concentration on what is being taught,

*“What I like is that I get to do other things at home when we are having classes online*, *like cleaning*, *cooking and cutting the grass*. *I can’t do this during face-to-face classes*, *so I like it now because it keeps me going*. *When I engage in many things*, *I don’t feel lonely and depressed*, *and this is how I am able to keep going during this COVID-19 crisis*. *The only thing is that you are not able to focus and give full concentration to the lecturer”*. (P81)

#### Family support

Support from family members was identified as an important facilitator to online learning as most of the students relied on their families to provide them with devices such as laptops and phones for their studies. Those who were able to access these devices enjoyed online learning,

*“As for me*, *what is still keeping me in the system is the help my parents and siblings give me every day because COVID-19 has really made things difficult for everyone in the world*. *My brother bought a laptop for me and that is what is helping me in my studies*. *Sometimes*, *I use my phone which my father gave to me so you can see that without support from my family*, *there is nothing I can do than to stop school in this COVID-19 times”*. (P82)

#### Counselling support

Regular counselling played a vital role in making students aware of the consequences of their decisions and choices during this uncertain times. For instance, it provided regular guidance to students who contemplated on halting their studies, subsequently, reducing anxiety and stress. One of the students explained her experience,

*“…What really helped me was when my mother made me talk to a counsellor when she saw that I was losing interest in my studies*. *In fact*, *I was finding it difficult coping with the online learning*, *and I was also afraid of COVID-19*. *It was becoming more stressful for me when my uncle had COVID and died but the counsellor helps to bring my stress down*. *She was encouraging me to try and continue my studies because nobody knows what will happen next year”*. (P112)

#### Seeking help

Many of students mentioned the ability to seek help from their lecturers and family members as an essential source of motivation for online learning. They sometimes contacted their lecturers for directives on their studies and in the process received encouragement, which kept them going. One of the students explained how the directives and words of encouragement from her lecturer, facilitated her studies,

*“The truth is in this time where people are dying from COVID-19*, *we all need people to show love and encourage us to keep moving because it is not easy for students*. *At a point I had wanted to drop my studies but some of my lecturers were very helpful and also my family members*. *They always tell us to continue to read and take our studies serious and that COVID-19 will soon go away*. *Two days ago*, *I called my lecturer to get more understanding on one of our assignments and this really encouraged me”*. (P1)

#### Frequent communication

According to majority of the students, communication was necessary to improve their online learning and maintain their well-being. Those who were able to communicate regularly with their lecturers were able to clarify relevant issues related to their studies and received the needed support. Regular communication with their peers also served as a support system, which increased their sense of belonging and minimized loneliness, fear and anxiety,

*“I will say that my biggest support and source of motivation is coming from my peers because I talk to them almost every day*. *When I do that*, *I feel I am not alone facing challenges in my studies because of COVID*. *When I begin to think like this*, *I don’t feel anxious and scared of COVID*. *I also try to get in touch with my lecturers when I need help in my studies*. *I send emails or viber messages and sometimes call them and this is helping me a lot”*. (P4)

#### Time management

Efficient time management was seen as an essential component of home-based learning. In order to meet their learning objectives, students recognized the need to identify specific times to engage in various activities such as home chaos and social media platforms and to avoid or minimize learning distractions,

*“Learning at home is not easy so if you are not able to manage and use your time properly*, *you cannot achieve your learning objectives*. *What has helped me so far is that*, *I make sure I don’t miss my online lectures because if you don’t take care you will replace the time for your online classes with other house chaos like cooking*, *eating*, *working in your garden and even chatting with friends on Facebook on viber*. *I plan my time very well so that I can read my lecture notes in the evenings before going to bed…”* (P38)

#### Control over learning space/environment

Students’ ability to have full control over their learning environment is necessary for effective online learning. To achieve this, they usually studied in quiet places, with less distractions and sometimes when their relatives were asleep,

*“…When it is time for my online class*, *I normally move to areas in my house not noisy and I tell my sisters not to come closer or talk to me until I am done*. *I make sure the place is quiet and nothing will interrupt my learning*. *My parents also understand the situation*, *so it is helping me to concentrate on my studies*. *Sometimes I have to learn at night when my siblings are all asleep to avoid interruptions”*. (P98)

The coping strategies employed by students in dealing with the online learning challenges included, families providing laptops and other relevant devices for online learning, emotional support from lecturers, counsellors and their peers through encouragement and effective communication. Having control over their learning environment and efficient management of their time, improved their focus and concentration on their studies. The flexibility in online learning and assessment made online learning less stressful.

## Discussion

The current study reveals COVID-19 misconception beliefs among students in the study area. While some of them considered COVID-19 pandemic as a natural occurrence, many perceived it as unreal, made by man to control population growth and as a means for the government to solicit for foreign aids. The misconceptions showed by the students in the study area, could be attributed to low awareness creation on the COVID-19 pandemic, which could subsequently affect their attitudes towards its control and prevention. Like other infectious disease, this could put students at risk of contracting COVID-19 and hinder their ability to learn. Previous studies have reported that denial of the existence, and misperceived causes of Ebola, HIV, Zika Virus and Yellow fever pandemics served as a set back to their preventive and control mechanisms, except when these were traced and addressed [45–48]. Similarly, misconceptions and myths on COVID-19 pandemic have contributed to the drastic spread of the disease in Ethiopia and Nigeria [49, 50]. Apart from the country and the healthcare system remaining vulnerable to the harmful impact of climate change and natural disasters such as cyclones, the economic and social impact of the pandemic could worsen the existing challenges faced by the country [51]. This necessitates the provision of additional relevant information to demystify the misconceptions, since this could reduce or control the spread of the disease in the study area.

This study found various challenges experienced by students in their online learning environment. Majority of the students regarded the home learning environment as a major challenge, particularly, because it affects their learning quality, emotional state, movement and interaction with their peers. These findings are consistent with other studies that have highlighted online learning challenge faced by students, during the COVID-19 pandemic, as psychosocial [52–55]. Our findings have also expanded on previous research to understand students’ online learning experiences by ascertaining the range of challenges linked to online learning.

Generally, the findings show variations in the challenges students face during online learning in relation to the availability of resources such as lack of computers and unstable internet service. Indeed, in the case of the pandemic, the movement restrictions, which made it impossible for students to visit school premises to access suitable learning facilities, and financial constraints could have contributed to aggravating the various challenges experienced by students. This is evident from the narratives discussed above, where students complained of their limited financial abilities to purchase laptops and other devices relevant to their online learning. Whilst other studies have identified common online learning challenges of students as the use of technology and competency [10, 14], this differed in a developing country like Fiji, as majority of the students regarded the home-based learning environment (distractions and interruptions from family members), and limited access to computers and internet services as their main challenges. This could be attributed to the fact that, a large proportion of the Fiji workforce is not in the formal sector and as such difficult to work from home during the lockdown, hence, loss of income. There was also loss of economic opportunities linked with seasonal work, more especially, because Fiji is a tourist dominated economy [56].

Additionally, students’ connecting their inability to acquire essential facilities such as computer, internet service and creating conducive learning space, to lack of financial resources, reveals the inequities within the education system, existing within countries. Studies have showed that, people in developing countries, who fall within the lower socio-economic levels have inadequate learning space at home and limited access to quality internet facilities [5].

Additional challenges identified included difficulty in acquiring practical skillset (lack of fieldwork, laboratory experiments and internships), feeling of anxiety, loneliness and stress, and limited interaction between students and their lecturers. Together, these had negative effect on the online learning experiences of students. This is in conformity with the previous studies on the adverse effect of lockdown on the learning experiences of students and challenges the home learning environment present [57, 58].

The feeling of loneliness, anxiety and stress experienced by the students in the study area, were attributed to lack of socialisation with their peers. Other scholars have established the negative effect of the COVID-19 pandemic on the emotional well-being of students [59, 60]. Challenges related to difficulties in accessing and using online platforms and ineffective tutorials experienced by students, call for the need to prepare them adequately for online learning as well as equipping lecturers with the necessary skills to undertake online tutorials effectively. There is also the need to exempt students from socio-cultural practices that hinder their effective learning. This study provides additional information on the need to pay attention to issues related to students’ emotional health, movement restrictions and their preparation for online learning.

Despite the healthcare advancement, recent data indicate unduly higher level of vulnerability rate amid the indigenous peoples [61, 62]. Studies have attributed this vulnerability to individuals having comorbidities, as well as low income that results from multiple families per dwelling, subsequently, aggravating infection risk [63]. These vulnerable groups include Fiji community, with rich culture and largely communal [64, 65]. Socio-cultural practices are of particular interest as compliance to the preventive measures of COVID-19 have been challenging in the Fiji community. This study findings further highlight the importance of understanding the impact of these socio-cultural practices on the knowledge and skills acquisition of students in the study area. Thus, apart from the noise interruptions to students, they sometimes missed their classes to attend these gatherings, amid the possibility of increasing the spread of the pandemic.

The learning challenges faced by students in the study area, highlighted above, could be attributed to the fact that, they were not prepared for the sudden transition from face-to-face learning to online. As such, most of them were still trying to adjust to the new practices of online learning. Relating this to the change management theory, the three phases, including unfreezing, changing and refreezing play a key role in managing the current transition [66]. The unfreezing of the traditional mode of learning was necessary to cater for unexpected situations from the COVID-19 pandemic. Thus, this was needed to control the spread of the pandemic and minimize the uncertainties in pursuing face-to-face classes. The unfreeze stage has provided an opening for students and relevant stakeholders to be inspired [67], as well as, providing a sense of safety to the learning community in Fiji Island, during the COVID-19 pandemic. The changing stage involves the ability of the institution (FNU), to innovate its own online learning modes for students or adopt a new one being used in other institutions.

The act of exploring appropriate methods for students, is a continuous process taking into consideration the concerns of students who are the beneficiaries of the change. Also, for any change to produce the expected results, issues such as time outlook and a new mindset should be considered [66]. Therefore, instead of students in the study area, paying less attention to online classes, by engaging in other activities, during lectures, they are rather expected to renew their minds and embrace the transition from face-to-face classes to online. This could enable them to search for ways to improve their online learning, thereby, incorporating resilience into the educational system [68]. The refreezing stage is inevitably, incorporating the use of technology in teaching and learning processes. Though this has become paramount in this COVID-19 crisis, it should be organised in a way that enable students to be able to adjust to the recent demands of technology, worldwide.

Nevertheless, students identified various coping strategies to overcome or minimise the impact of the challenges encountered during online learning. Majority of them relied on the support from their families to overcome challenges related to equipment (computers and internet access) and learning environment. Those without personal laptops borrowed from their relatives for their online classes. In order to improve the suitability of their home learning environment, they moved to places that were quiet, talked to their relatives to keep quiet and sometimes learned at times when family members were asleep. This was what one of the students (P98), highlighted by saying “*sometimes I have to learn at night when my siblings are all asleep to avoid interruptions”*. Providing suitable learning environment could improve learning quality and skills acquisition.

The difficulty encountered by accessing online learning platforms including Moodle, was overcome through encouragement and seeking assistance from lecturers and their peers. Studies have recommended the need to provide appropriate guidelines for students as well as training on current technologies for online learning [4]. Issues related to mental and emotional health of students (anxiety, stress and loneliness), were improved through frequent communication with their peers, families and seeking counselling support where necessary. Given that students’ mental health was affected by the uncertainty of the COVID-19 pandemic, the work of educational counsellors has been regarded very essential [69].

Although, students regarded flexibility associated with online learning and assessments as their coping strategies, these can compromise the quality of knowledge and skills acquisition, especially, where students decide to copy examination answers from online portals. Like this study, other scholars have identified flexibility, increased comfort and convenience as advantages of online learning [70].

Proper time management and priority setting are essential components of online learning among students to increase concentration during online classes and to accomplish assignments on time. A student’s decision to embrace any of the strategies highlighted above, could be a complex interplay between factors such as the availability of resources, family organization, the personality of the student and the level of connection with their lecturers and peers, and capacity.

### Study implications

The findings of this study have many implications. Firstly, this study has highlighted the essence of understanding the misperceived causes of COVID-19, for appropriate interventions. Secondly, the study findings have provided an understanding on the various challenges FNU students faced due to the abrupt shift to full online learning. The critical areas that need immediate attention were poor internet services, inadequate home learning environment, lack of learning devices and difficulties accessing online platforms and resources (Moodle, library and books). Though we do not intend to generalise the findings of this study due to the research methods used, the findings could be useful to schools with comparable learning setting to develop and improve their individual continuity strategies to lessen the adverse effect of the COVID-19 pandemic. In addition, the findings of this study provide students with essential information on the potential strategies that they may use in overcoming various online learning challenges. Lastly, lecturers, policymakers and relevant bodies within the university setting, may use the findings to provide appropriate interventions to address the various challenges faced by students.

## Strengths and limitations

The limitation of the study relates to our inability to generalize the findings due to the use of purposive sampling in selecting participants for the study. Generalization of the findings is not possible because the perceptions and experiences of individuals may differ from one place to another. However, this study has provided detailed and contextual information on COVID-19 misconception beliefs, challenges associated with online learning as well as their coping strategies. Additional research is needed to understand lecturers’ teaching experiences to obtain a holistic view of the situation.

## Conclusion

Migrating fully to online learning during the COVID-19 pandemic is serving the purpose of education continuity, including teaching and learning in the study area. However, to improve effective learning among students, the challenges they face in relation to online learning should be addressed by the relevant authorities. Though students regarded their ability to engage in other tasks during online classes, and easy assessment processes, as their coping strategies, these can have adverse effect on their studies and create knowledge gap. Considering the challenges associated with the Covid-19 pandemic, it is anticipated that, students would be searching for various means of relaxation and comfort, but this should not compromise the quality of their studies, hence the need for a change management programme to address the situation. Also, relevant information should be provided to students to correct COVID-19 misconceptions, since this could reduce its spread.

## Supporting information

S1 File Semi-structured interview guide. (DOCX)

S2 File Thematic mapping diagram. (JPG)

S3 File (DOCX)
