# Supplementary material for: What do university students say about online learning and the COVID-19 pandemic in central Fiji? A qualitative study
Source: PLoS One. 2022 Aug 23;17(8):e0273187. doi: 10.1371/journal.pone.0273187 (PMC9592056; doi:10.1371/journal.pone.0273187)
Supplement: S1 File — (DOCX) [file pone.0273187.s001.docx]

**Semi-structured Interview Guide**

(Expected time duration 30-45 minutes)

**Social Demographic Characteristics**

1. Respondent’s unique identification number:
2. Age (completed years):
3. Gender:
4. Ethnicity:
5. Religion:
6. marital status:
7. College:
8. Programme (bachelor, master etc):
9. Interview start time:
10. Interview end time:
11. Interview date:

**Interview Comments:** This includes recording the interview venue, study participant mode, expressions (including non-verbal) and interactions that could contribute to providing in-depth understanding of the context of the interview.

**Questions/Probes**

1. What do you think is the cause of COVID-19?
2. Do you think COVID-19 has in anyway affected your health and wellbeing? *(If not mentioned, probe to find out about issues related to stress, depression, anxiety, loneliness).*
3. Do you think COVID-19 has affected your learning in any way? If yes how, if no why?
4. What are your experiences regarding online learning during the COVID-19 pandemic?
5. What are the challenges you face during online learning?
6. What are your likes and dislikes about online learning during the pandemic?
7. What are the strategies you have put in place to overcome your online learning challenges?
