# Supplementary figures and images for: What do university students say about online learning and the COVID-19 pandemic in central Fiji? A qualitative study
Source: PLoS One. 2022 Aug 23;17(8):e0273187. doi: 10.1371/journal.pone.0273187 (PMC9592056; doi:10.1371/journal.pone.0273187)

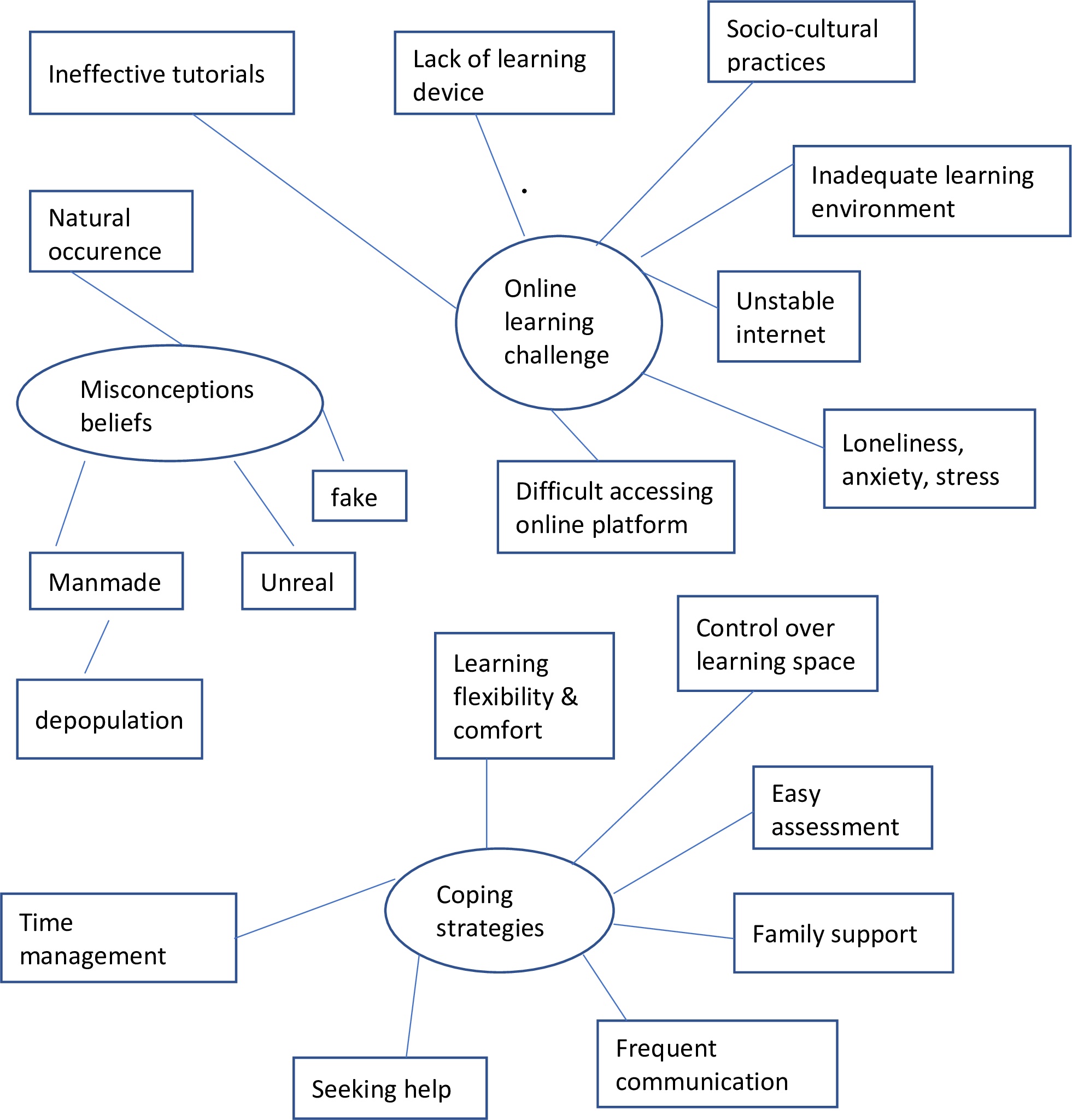

Supplement: S2 File — (JPG) [file pone.0273187.s002.jpg]
