# Supplementary material for: What do university students say about online learning and the COVID-19 pandemic in central Fiji? A qualitative study
Source: PLoS One. 2022 Aug 23;17(8):e0273187. doi: 10.1371/journal.pone.0273187 (PMC9592056; doi:10.1371/journal.pone.0273187)
Supplement: S3 File — (DOCX) [file pone.0273187.s003.docx]

I: Can you tell me about the cause of this COVID-19 pandemic?

P70: I will say that COVID-19 is something that has happened naturally, and we cannot put the blame on anybody. It’s difficult to explain some of these things so I will say God knows it all. As human beings we will talk about virus causing COVID-19, which is ok but this is not straight forward. Is like every 100 years something like this happens. I learnt about 100 years ago, there was another pandemic that wiped people from the surface of the earth. This is sad but only God knows best (22 years old).

I: In your opinion what caused the pandemic? Please tell me everything you know about it.

P7: In my point of view, I believe COVID-19 was the biggest scam of all times. It was a manmade virus, created by the secret societies for their depopulation agenda and to put fear in humans. For some years now, I have been researching about the secret society. They are known to many as the illuminati or the Free Mason, but these are just small societies who dance to the tunes of the elites. So from there, I learnt of their agenda and one of it is the virus that’s affecting everyone in the world today. If you are not awake than you won’t believe what I’m telling you.

I: What are your views on the causes of COVID-19.

P20: If you ask my friends about my view towards this plandemic or scamdemic, or for the coronavirus, they will tell you that I think that it’s all fake. So COVID-19 was planned to happen many years ago and the elites are those spreading the news in the media. You should try and watch the opening ceremony of the 2012 Olympics. The elites have a lot of things to do and this pandemic is the beginning of it. Worse is yet to come.

I: Please so what brought about this pandemic that we are all fighting now?

P17: To be honest, I just continue with my normal routines. I believe that the Fiji government is just manifesting what the people want. I believe there is no virus in Fiji right now. This is all just a show, to get aid from overseas, so they can make money. There is no Indian variant here. Everyone is a joke.

I: Please tell me what you know about the cause of COVID-19 pandemic.

P34: I feel sorry that we are being used as a scape goat because I don’t believe there is any virus. The government is using it to get money from developed countries. The time is coming when people will know the truth, and the worst part is the media, the biggest liar, making us believe in what they tell us, which is not true.

I: Do you think COVID-19 has affected your learning in anyway?

P3: Yes, COVID-19 has affected my usual way of learning. I am now having online classes, which is difficult for me especially attending discussions and tutorials. I feel tired on a day-to-day basis and bored sometimes. It is a lot of work learning online but yes, these restrictions by the cruel and greedy government are affecting every student in Fiji and there is nothing we can do about it.

I: What are the challenges you face during online learning in this pandemic?

P56: I think our lecturers and the people running the school should do something about online tutorials and make it enjoyable like the face-to-face tutorials. I don’t like the online tutorials at all. It’s very boring and not interesting. I learn a lot from during the face-to-face tutorials but now everything is online, and things are not working properly. Is a big problem but what can we do.

I: Can you please mention the online learning challenges you facing since the pandemic began?

P85: I don’t have a phone at the moment so it’s hard keeping up with zoom lectures. I don’t also have a computer or iPad. There is no money to buy a computer since my father lost his job because of COVID so tell me how a student will be able to engage in online classes without these things. I borrow from my friend or cousin, but I can only get it when they don’t have a class. This situation is a serious one so we need help from the university and the government so that we can cope with this online learning.

I: So, what will you say are the issues affecting your studies now?

P73: It is very difficult keeping up to date with discussions and tutorials plus zoom lectures. Sometimes, my brother’s classes clash with mine, so I just sacrifice because it is not my laptop. So, I can say my challenge is not having a phone and a laptop. It is very hard for me. I find it easier with face-to-face learning.

I: Yes, you can go ahead and mention some of your online learning challenges.

P15: It’s like a first class learning and although I feel for the students who cannot afford data and sometimes too the internet is not good, so it goes on and off during online classes. Internet is affecting our studies When the internet becomes slow, you cannot even download articles and books to read.

I: How does this affect online learning?

P9: I will say the COVID-19 is causing more harm to students because it has made learning very difficult. This is why I like the face-to-face classes but because of COVID-19, we are all doing online learning and this is too difficult. Sometimes I even find it difficult to access my online quiz and upload assignments on Moodle and also when I am searching for papers and books from the library online. As I said I like the face-to-face classes more than the online because its straightforward.

I: So, what other challenges are you facing?

P36: The truth is that I don’t feel part of my friends during online classes. You just feel like you are alone and isolated from everybody. That connection is not there so I am not interested in online classes at all. As a student you just want to meet with your friends on campus and feel that you are also part of them not online. Before the COVID, we sometimes shared ideas during group discussions, and we were there for each other in many ways, but we cannot do that now.

I: What are the other challenges you are talking about? Do you want to talk about them?

P49: I know some of my friends are happy learning at home, but I am not happy because I am not able to focus very well. The best thing is for me to rent my own apartment, but I have no money for that so I’m sleeping in the room with my two sisters. Sometimes I don’t even get the time to sit and work on my assignments and do my online quizzes on time.

I: What are your online learning experiences?

P83: When you are home, you get many distractions such that you don’t even know when is the good time and place to sit and learn. Sometimes, I work in my farm and after that get tired that I even forget to read and miss my online classes. I will say home is not the good place to learn because when I go to campus, I can sit in the library and other places to read and if I don’t understand anything, I can talk to some of my lecturers and classmates.

I: What has been some of the challenges with the online learning?

P97: I will say the online learning has many challenges, affecting students. Just consider somebody like me who is taking courses that need fieldwork. How can I complete my studies in agriculture without going to the farms to get practical experience? So you will agree with me that the online learning is not helping us at all but what can we do now that COVID-19 has taken over the system.

I: What don’t you like about online learning?

R55: The main challenge we are facing as students doing health courses is that we are not able to do practicals like lab tests and go for internships. These things are not possible because COVID-19 has restricted our movements throughout the country, but I learn a lot during practical sessions. Online learning is good, but it can only provide theories not practicals..emmh, yeah, I will say some of us are just not happy with this online thing. I need to get the practical skills to build my career.

I: What are your online challenges?

P18: I will say that the religious activities going on in my house is affecting my studies and I cannot do anything about it because my father leads people to pray and perform other rituals at home for protections and other things. Few weeks ago, we came together to celebrate our festival. Now because of the COVID restrictions many people don’t come to the house to pray but the extended family members and close friends come over and they make a lot of noise, which makes it difficult for me to attend online classes. Apart from the noise, I have to also attend these events, so I sometimes miss my classes.

I: How are you managing your online learning challenges?

P22: Yeah I will say ehm, I think it is a good strategy, getting students to engage in online lectures, tutorials and more. Students are enjoying it because some just turn off their cameras from home and just listen to the lecture while having breakfast or lying down. I sometimes do other things in the house like cooking and at the same time listening to my lecture.

I: Can you tell me some of strategies you are using?

P23: One good thing about COVID-19 is that, now we don’t write normal exams like sitting in a big hall with somebody watching over us so that we don’t copy from each other or make noise, which is difficult. Now it’s easy because everything is online. You can easily copy the answers of your test questions from google, which makes things easy for students.

I: What do you like about the online learning?

P81: What I like is that I get to do other things at home when we are having classes online, like cleaning, cooking and cutting the grass. I can’t do this during face-to-face classes so I like it now because it keeps me going. When I engage in many things, I don’t feel lonely and depressed, and this is how I am able to keep going during this COVID-19 crisis. The only thing is that you are not able to focus and give full concentration to the lecturer”.

I: Can you tell me some of the strategies you are using to overcoming some of your online learning challenges?

P82: As for me, what is still keeping me in the system is the help my parents and siblings give me every day because COVID-19 has really made things difficult for everyone in the world. My brother bought a laptop for me and that is what is helping me in my studies. Sometimes, I use my phone which my father gave to me so you can see that without support from my family, there is nothing I can do than to stop school in this COVID-19 times.

I: What are some of your coping means?

P112*:* We all know online learning is not easy but hmm, what really helped me was when my mother made me talk to a counsellor when she saw that I was losing interest in my studies. In fact, I was finding it difficult coping with the online learning, and I was also afraid of COVID-19. It was becoming more stressful for me when my uncle had COVID and died but the counsellor helps to bring my stress down. She was encouraging me to try and continue my studies because nobody knows what will happen next year”.

I: So, if I may know how you are managing the situation because you have mentioned that online learning is not easy.

P1: The truth is in this time where people are dying from COVID-19, we all need people to show love and encourage us to keep moving because it is not easy for students. At a point I had wanted to drop my studies but some of my lecturers were very helpful and also my family members. Hmmm they always tell us to continue to read and take our studies serious and that COVID-19 will soon go away. Two days ago, I called my lecturer to get more understanding on one of our assignments and this really encouraged me.

I: What strategies are you using to manage the situation?

P4: I will say that my biggest support and source of motivation is coming from my peers because I talk to them almost every day. When I do that, I feel I am not alone facing challenges in my studies because of COVID. When I begin to think like this, I don’t feel anxious and scared of COVID. I also try to get in touch with my lecturers when I need help in my studies. I send emails or viber messages and sometimes call them and this is helping me a lot.

I: Please tell me what you have in place to overcome your online learning challenges.

P38: Learning at home is not easy so if you are not able to manage and use your time properly, you cannot achieve your learning objectives. What has helped me so far is that I make sure I don’t miss my online lectures because if you don’t take care you will replace the time for your online classes with other house chaos like cooking, eating, working in your garden and even chatting with friends on Facebook on viber. I plan my time very well so that I can read my lecture notes in the evenings before going to bed. Ehmm we all must manage Covid-19 issues because this is where we are now in life.

I: How do you deal with the challenges you face during online classes.

P98: When it is time for my online class, I normally move to areas in my house not noisy and I tell my sisters not to come closer or talk to me until I am done. I make sure the place is quiet and nothing will interrupt my learning. My parents also understand the situation so it is helping me to concentrate on my studies. Sometimes I have to learn at night when my siblings are all asleep to avoid interruptions.
